# Testosterone response to competition in males is unrelated to opponent familiarity or threat appraisal

**DOI:** 10.3389/fpsyg.2014.01240

**Published:** 2014-11-03

**Authors:** Gonçalo A. Oliveira, Sara Uceda, Tânia F. Oliveira, Alexandre C. Fernandes, Teresa Garcia-Marques, Rui F. Oliveira

**Affiliations:** ^1^Unidade de Investigação em Eco-Etologia, ISPA – Instituto UniversitárioLisbon, Portugal; ^2^Laboratorio de Psicobiología, Departamento de Psicología Experimental, Universidad de SevillaSeville, Spain; ^3^Unidade de Investigação em Psicologia Cognitiva, do Desenvolvimento e da Educação, ISPA – Instituto UniversitárioLisbon, Portugal; ^4^Champalimaud Neuroscience Programme, Instituto Gulbenkian de CiênciaOeiras, Portugal

**Keywords:** androgens, testosterone, challenge hypothesis, sex factors, cognition

## Abstract

It has been proposed in the literature that the testosterone (T) response to competition in humans may be modulated by cognitive variables. In a previous experiment with a female sample we have reported that opponent familiarity and threat appraisal moderated the T response to competition in women. With this experiment we aim to investigate if these variables have the same impact on males T response to competition, extending the previous findings in our lab. Forty male participants (20 dyads) were recruited to engage in a same sex, face to face competition using the Number Tracking Test as a competitive task. Levels of T, cortisol (C) and dehydroepiandrosterone (DHEA) were measured before and 20 min after the competition. Results show that losers report higher levels of threat than winners and increased their T levels after the competition, however this T change was not predicted by opponent familiarity or threat appraisal. No variation was detected for C and DHEA levels. These findings suggest that there could be sex differences for the moderators/mediators of the T response to competition in humans.

## INTRODUCTION

Androgen responses to social challenges are present in several taxa and have been interpreted as a mechanism to adjust the internal state and the output of androgen dependent behaviors to changes in the social environment (Oliveira, 2009). Early explanations for this response stressed the reciprocal relationship between androgens and behavior (e.g., Leshner, 1975) and culminated in the formalization of two independent hypotheses for the social modulation of androgens: the biosocial model (Mazur, 1985) and the challenge hypothesis (Wingfield et al., 1990).

The biosocial model (Mazur, 1985; Mazur and Booth, 1998) postulates a mutual reinforcing relationship between androgens and dominance. Androgens, testosterone (T) in particular, promote status-seeking behaviors and when high status is achieved, in an agonistic interaction, the individual’s androgen levels increase to match the new position in the social hierarchy. On the contrary, after losing a competition T levels are expected to decrease, to avoid the possible status costs of further contests. In the “challenge hypothesis” (Wingfield et al., 1990), transient changes in androgen levels adjusts the expression of androgen-dependent aggressive behaviors to the social context, thus avoiding the costs associated with keeping chronically elevated T levels. Although initially proposed in birds, the “challenge hypothesis” has been extended to other taxa including humans (Archer, 2006; Hirschenhauser and Oliveira, 2006). In response to an agonistic interaction, the “challenge hypothesis” predicts an increase in T levels without specifically defining if this effect is valid for winners and losers. However, neither of the these two hypotheses explain the diversity of T responses to competition in humans found in the literature (Oliveira and Oliveira, 2014b). In recent reviews, this variety of androgen responses to social competition has been interpreted as a consequence of the moderation of the androgen response by cognitive variables, with appraisal emerging as the strongest candidate for this effect (Salvador and Costa, 2009; Oliveira and Oliveira, 2014b).

Appraisal can be defined as a continuous evaluation process of the transactions between the individual and the environment, in which the individual assesses the significance and the implications of an event (Scherer, 2001, 2009). Therefore, the appraisal of an event results from an interaction in which the objective structure of the event is contrasted with the goals, resources and abilities of the individual. In goal relevant situations (e.g., competitive contexts), appraisal can be understood within a demands/resources continuum (Tomaka et al., 1997; Blascovich and Mendes, 2000). When an individual evaluates the demands of a task as exceeding the available coping resources, the situation will be appraised as a threat. In contrast, if the perceived resources exceed the task demands, the event will be evaluated as a challenge. In addition to affective and cognitive differences between threats and challenges, patterns of cardiovascular response activated in states of task engagement are specific to each type of appraisal (e.g., threat: lower cardiac reactivity, increased vasoconstriction; challenge: high cardiac reactivity, lower vasoconstriction; for a review see Blascovich et al., 2011). Some studies have already provided data that supports the role of appraisal in the androgen responsiveness to social competition. For example, male cichlid fish (Mozambique tilapia, *Oreochromis mossambicus*) fighting unsolved fights against their own image on a mirror (i.e., where they do not experience either a victory or a defeat) fail to exhibit an androgen response, despite expressing similar levels of aggressive behavior to those of males fighting a real opponent (Oliveira et al., 2005; Hirschenhauser et al., 2008). This dissociation between behavior and androgen response can be explained by the different evaluations the subject makes of unsolved fight and of fights with perceived positive or negative outcomes (Oliveira, 2009). Human studies have also provided evidence for the role of appraisal on the androgen response. For example, a laboratory experiment reported that the T response to a face-to-face competition was higher when the opponent was evaluated as having high self-efficacy (van der Meij et al., 2010).

Familiarity is one of the first components to be evaluated in the appraisal process (Scherer, 2001) and this variable has been extensively studied in the context of agonistic encounters with non-human animals. It has been described, for several territorial species of different taxa, that in aggregated stable territories familiar opponents (e.g., neighbors) pose less threat and elicit less aggression than unfamiliar individuals (dear enemy effect; Ydenberg et al., 1988; Temeles, 1994), while in other species neighbors are more likely to compete for territory and mates and thus elicit a higher aggressive response than roaming strangers (Müller and Manser, 2007). In humans, effects of familiarity in social challenges have also been described in the literature. In a domino team competition, T tended to increase more when players were facing teams that were not from their own village (Wagner et al., 2002). Also, ingroup membership has been suggested as an explanation for the lack of T response in a sports competition (Trumble et al., 2012) and elicited different T responses for high ranked players in a video game competition (Oxford et al., 2010).

In a previous experiment with women, we provide stronger evidence that familiarity moderated T responses to a face-to-face competition event appraised as a threat (Oliveira et al., 2013). In this study T increased more in losers that evaluated the outcome as a threat while competing against unfamiliar opponents, while cortisol (C) and dehydroepiandrosterone (DHEA) levels remained at their pre-competition levels (Oliveira et al., 2013). On the other hand, winners appraised the competition outcome as less threatening than losers and no significant changes were detected for any of the measured hormones (T, C, and DHEA). Because men and women tend to exhibit differences in appraisal tendencies toward competition (for a review see Niederle and Vesterlund, 2011) and it has been previously suggested that there may be sex differences for the T response to competition (Kivlighan et al., 2005; Josephs et al., 2011), we decided to investigate if the previous findings described above would also be valid for males, or if there was a sex difference in the cognitive moderation of the T response to competition in humans. Therefore, in this study we tested if males display the same pattern of endocrine response as females to a face-to-face contest, and if opponent familiarity and threat vs. challenge appraisal of the outcome (winner/loser) moderates males T response to competition, using the same experimental paradigm as in Oliveira et al. (2013). Although we have not found changes in C and DHEA for women, these hormones were also monitored in this experiment since it is established that C responds to social stress, influences cognitive variables (e.g., McEwen and Sapolsky, 1995) and is known to interact with T in case of social contests (Mehta and Josephs, 2010). On the other hand, DHEA is an important androgen involved in the regulation of aggressive behavior (Soma et al., 2014) and on the processing of threat signals in humans (Sripada et al., 2013).

## MATERIALS AND METHODS

### PARTICIPANTS AND EXPERIMENTAL PROTOCOL

Forty undergraduate psychology male students (24.00 ± 6.99 years) voluntarily signed up to participate in experimental sessions that lasted for approximately 1 h. To control for circadian variation of hormone levels all sessions were scheduled for the afternoon (12:30–17:30). Participants were tested in dyads (*n* = 20). One participant presented a pre-competition level of T above 3 standard deviations and therefore its pair was excluded from the sample, bringing the total number of participants to 38 (19 dyads). All participants were rewarded with one course credit and received a monetary payment depending of their condition (winners: 8€, losers: 4€). A male and a female experimenter were present in all the experimental sessions. This experiment was performed in accordance to Portuguese regulations, the declaration of Helsinki and with the approval of the ethics committee of ISPA’s Research Center. Written consent was given by all participants.

### DATA COLLECTION AND PSYCHOLOGICAL VARIABLES

Participants were asked to sit face-to-face across a table. An opaque vertical barrier was placed on the top of the table between the participants, such that it enabled the participants to establish eye contact but blocked the view of the opponent task and questionnaires during the experiment.

At the beginning of the experiment participants were asked to provide a pre-competition saliva sample and filled in a questionnaire that controls for possible sources of hormone variation. After completing this questionnaire, pairs were asked to rate from 1 to 5 how familiar they were with each other prior to this experiment (1 = not familiar; 5 = very familiar). Instead of classifying the pairs as “familiar” vs. “not familiar,” we have used a continuous measure since it better matches familiarity as a signal-detection component of appraisal (Scherer, 2001).

As in previous experiments (Schultheiss et al., 1999; Carré et al., 2009; Oliveira et al., 2013), the Number Tracking Test (NTT) was used for the competitive task. The NTT requires participants to connect a sequence of consecutive ascending numbers (1-, 2-, 3-, 4-, …) arranged in a matrix and surrounded by distracting numbers, until a highlighted number is reached. To experimentally assign participants to the winner or loser condition, the length of the NTT matrices was manipulated (i.e., winners had shorter NTT matrices than losers). This procedure has been previously used in NTT competition (Schultheiss et al., 1999; Carré et al., 2009; Oliveira et al., 2013) and allows an undetectable manipulation of the outcome, since participants have no access to their opponent matrices and therefore cannot assess the relative difficulty of their matrices. Experimental conditions associated with a side of the table were randomized and pre-determined before the experiment and participants were free to choose their position. Instructions were focused on the competitive nature of the task and it was also highlighted that the participants would compete against one another over 12 NTT trials and receive 1€ for each trial they had won up to a maximum of 12€. Feedback about who was the first to reach the highlighted end number on each NTT matrix characterized a trial as a “Win” or a “Loss” to the participant. The outcome was confirmed by the experimenter on each trial and 1€ was immediately given to the winner. This was done in order to reinforce the authenticity of the result and the competitive nature of the task.

After the completion of a NTT matrix for training, participants competed over three sets, each one composed by four NTT matrices. The matrices on the first and second NTT sets were manipulated to create a draw between the participants (four wins, four losses). The third NTT set defined the outcome of the competition with the participant in the winner condition winning the four NTT trials and the participant in the loser condition losing the four NTT trials. The outcome of the competition for two pairs was not congruent with the assigned condition (i.e., participant assigned the winner matrix lost the competition) and since their exclusion did not alter the main results reported here, they were coded to their real outcome and included in the sample.

After the competition, participants were asked to evaluate the outcome as a threat and as a challenge using two items with a 4 points scale as in our previous study (Oliveira et al., 2013). Personality questionnaires unrelated to this experiment were given to the participants as a filler task for 20 min, until they were asked for a second saliva sample.

### HORMONE ASSAYS

Participants were instructed to abstain from smoking, eating, drinking, physical exercise, brushing their teeth or consuming pH altering substances (several examples for this option were included) for 1 h before the experiment. Saliva samples were collected by passive drool into 5 ml polypropylene vials and stored at -20°C right after the end of the experiment. Samples were thawed, centrifuged at 2245 *g* for 10 min and the supernatant stored at -20°C until the assay. Luminescence Immunoassay kits (IBL, Hamburg, Germany) were used to determine concentrations of free T, C, and DHEA. The intra-assay and inter-assay coefficients of variance were respectively 6.1 and 8.6% for T, 8.3 and 12.4% for C, and 4 and 11.9% for DHEA. Absolute values for all measured hormones are presented in **Table 1**.

**Table 1:** Baseline and post-competitive hormone levels for Winners and Losers.

		Baseline mean (±SE)	Post-competition mean (±SE)
Winner	T (pg/ml)	146.875 (±13.020)	157.400 (±16.299)
	C (ng/ml)	3.892 (±0.609)	4.646 (±0.729)
	DHEA (pg/ml)	739.355 (±107.281)	563.953 (±49.523)
Loser	T (pg/ml)	120.167 (±15.027)	150.814 (±16.054)
	C (ng/ml)	2.952 (±0.466)	3.631 (±0.406)
	DHEA (pg/ml)	512.576 (±68.289)	633.585 (±55.824)

### PRELIMINARY ANALYSIS

A skewed distribution was found for the C levels and therefore these measures were log-transformed before statistical analysis. No transformation was required for T or DHEA levels. All measures were scanned for 3 standard deviation outliers and as reported before, one pair was excluded from the sample. Familiarity between opponents was measured and not manipulated. Ratings for familiarity ranged from 1 to 5 [mean ± standard error of the mean (SEM) = 2.68 ± 1.454].

### STATISTICAL ANALYSIS

Pairs of competitors were compared using a mixed model analysis of covariance (ANCOVA) with Outcome (winner, loser) as a within subjects factor, Familiarity as a covariate and each dependent variable as a repeated measures factor. The repeated measures factor tested in different ANCOVA models were: Appraisal (threat, challenge) and the measures for T, C, and DHEA (pre-, post-competition). Planned contrasts were used for *a priori* comparisons and therefore the reported degrees of freedom match those of the ANCOVA model. Degrees of freedom vary for the DHEA statistical analysis due to an insufficient volume of saliva to run this hormone assay for two of the participants. Partial eta squared ($\eta_{p}^{2}$) effect sizes are provided for main effects and interactions. Effect sizes for contrasts were calculated using Cohen’s *d* with the average of standard deviations as the standardizer and converted to Hedge’s *g* corrected for sample size bias (Lakens, 2013).

For the moderation analysis (Aiken and West, 1991), the unstandardized residuals from regressing the pre-competition T on post-competition T, were used as an index of T response and inserted as the dependent variable. The variables threat and familiarity were used as predictors and the interaction term was calculated as the product of threat by familiarity.

## RESULTS

### APPRAISAL OF THE COMPETITION OUTCOME AS THREAT AND CHALLENGE

**FIGURE 1:**
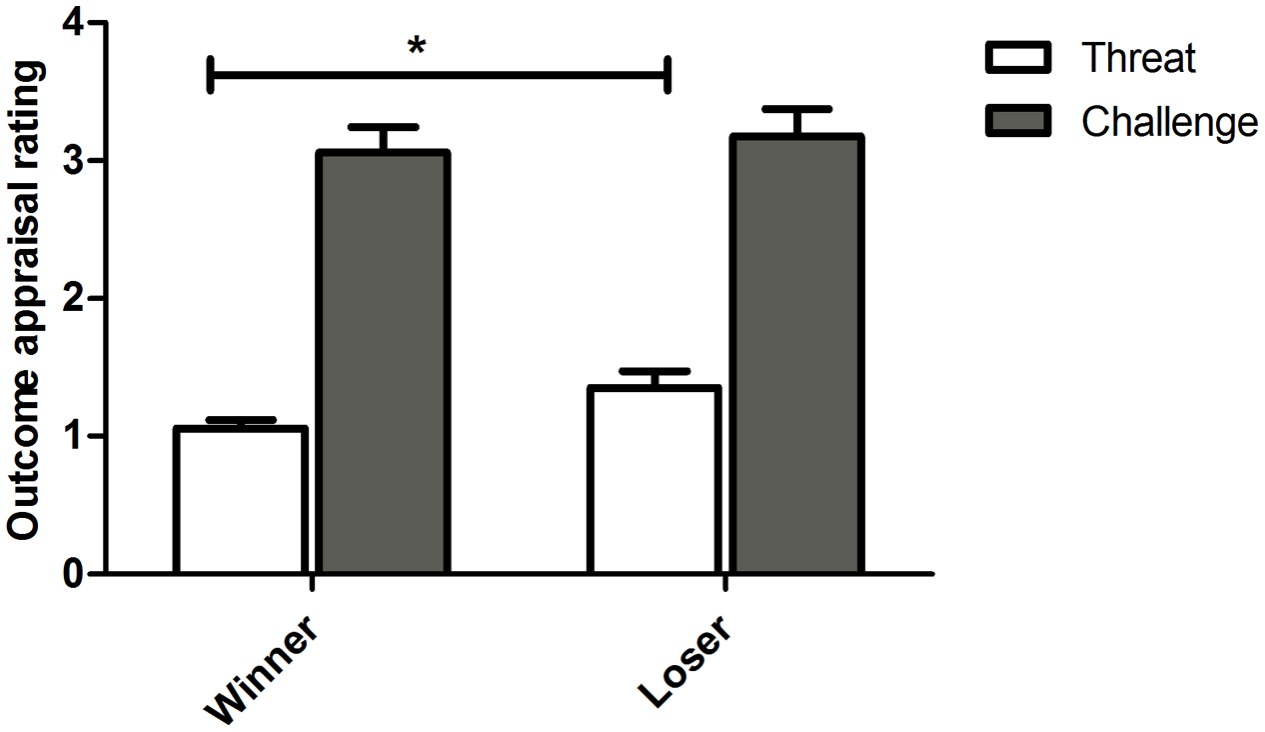
**Competition outcome appraisal rating as a Threat/ Challenge (Mean ± SEM) for participants in the winner and loser condition with familiarity of the opponent as a covariate.** Asterisk indicates significant differences at *p* ≤ 0.05.

Overall, participants rated the outcome as more of a challenge than a threat [**Figure 1**; *F*(1,15) = 48.856, *p* < 0.0001, $\eta_{p}^{2}$ = 0.765]. Losers appraised the competition outcome as more threatening than winners [*t*(15) = 2.114, *p* = 0.051, *g* = 0.781]. For challenge appraisal, no differences were found between the conditions [*t*(15) = 0.404 *p* = 0.691, *g* = 0.147]. No familiarity effects were detected on the evaluations as threat and challenge (all β n.s.; Threat/Challenge × Outcome × Familiarity: *F*(1,15) = 0.845, *p* = 0.372, $\eta_{p}^{2}$ = 0.053).

### HORMONAL VARIABLES

**FIGURE 2:**
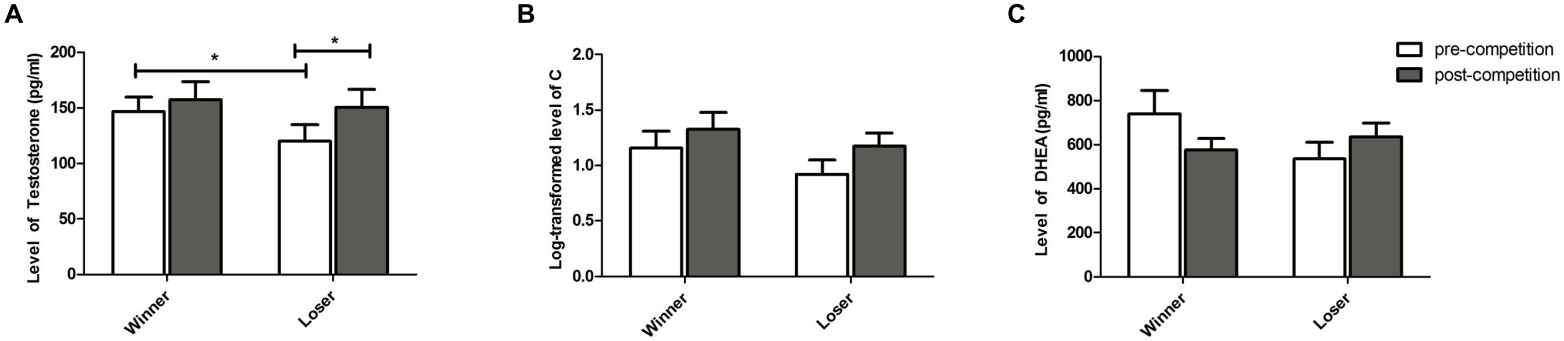
**Hormone levels (Mean ± SEM) measured at baseline (pre-competition) and 20 min after the competition (post-competition) for participants in the winner and loser condition with familiarity of the opponent as a covariate. (A)** testosterone, **(B)** cortisol, and **(C)** DHEA. Asterisk indicates significant differences at *p* ≤ 0.05.

*Testosterone* (**Figure 2A**) – No overall variation of T levels was detected over the competition [*F*(1,17) = 0.004, *p* = 0.946, $\eta_{p}^{2}$ < 0.001]. The two treatments showed different responses to the competition, with a significant increase in T in losers [*t*(17) = 2.601, *p* = 0.018, *g* = 0.442] and no significant change detected in winners [*t*(17) = 0.853, *p* = 0.405, *g* = 0.060], T increased in losers. Winners and losers showed different pre-competition levels of T, with subsequent winners exhibiting higher levels than subsequent losers [*t*(17) = 2.609, *p* = 0.018, *g* = 0.427]. However, no differences in T levels were found between winners and losers at the end of the competition [*t*(17) = 0.498, *p* = 0.624, *g* = 0.091]. No effects were detected for the covariate familiarity on the T levels [all β n.s.; T × Outcome × Familiarity: *F*(1,17) = 0.232, *p* = 0.636, $\eta_{p}^{2}$ = 0.013].

*Cortisol* (**Figure 2B**) – There was no overall variation of C levels over the competition [*F*(1,17) = 1.951, *p* = 0.180, $\eta_{p}^{2}$ = 0.102] and there were no differences between the two treatments either before [*t*(17) = 1.220, *p* = 0.239, *g* = 0.373] or after the competition [*t*(17) = 0.785, *p* = 0.443, *g* = 0.249]. Within each treatment, there was no C variation over the competition in winners [*t*(17) = 1.106, *p* = 0.283, *g* = 0.246] and a only a marginal increase was observed in the losers [*t*(17) = 1.906, *p* = 0.073, *g* = 0.461]. Furthermore, no significant effects of familiarity were found [all *β* n.s.; C × Outcome × Familiarity: *F*(1,17) = 0.579, *p* = 0.456, $\eta_{p}^{2}$ = 0.032].

*Dehydroepiandrosterone* (**Figure 2C**) – No overall changes of DHEA levels over the competition were detected [*F*(1,15) = 1.685, *p* = 0.213, $\eta_{p}^{2}$ = 0.101] and DHEA levels were not different between the two treatments either before [*t*(15) = 1.698, *p* = 0.109, *g* = 0.530] or after the competition [*t*(15) = 0.670, *p* = 0.512, *g* = 0.247]. Winners marginally decreased their levels of DHEA after the competition [*t*(15) = 1.963, *p* = 0.068, *g* = 0.485] and no changes in DHEA were detected for losers [*t*(15) = 1.183, *p* = 0.254, *g* = 0.344]. Furthermore, no significant effects of familiarity on DHEA levels were found [all β n.s.; DHEA × Outcome × Familiarity: *F*(1,15) = 0.105, *p* = 0.750, $\eta_{p}^{2}$ = 0.006].

### ASSOCIATION BETWEEN HORMONES AND PSYCHOLOGICAL VARIABLES

No significant association between familiarity, appraisal and the post-competitive hormones levels were detected either for winners or for losers (**Table 2**).

**Table 2:** Pearson correlation coefficients between threat, familiarity and hormone levels 20 min after the competition for Winners (*n* = 18) and Losers (*n* = 18).

		Threat	Familiarity	T2	C2	DHEA2
Winner	Threat	1	-0.308	0.320	-0.053	0.150
	Familiarity	-0.308	1	0.067	0.138	0.192
Loser	Threat	1	0.276	0.113	-0.069	0.160
	Familiarity	0.276	1	0.003	-0.218	-0.196

### MODERATION ANALYSIS

The regression model predicting T variation as a function of Threat, Familiarity and Threat × Familiarity was not significant (*R*^2^ = 0.105, *p* = 0.658). Both predictors and the interaction term were also not significant (All Threat: β = 0.367, *p* = 0.299; Familiarity: β = 0.206, *p* = 0.232; Threat × Familiarity: β = -0.134, *p* = 0.372).

## DISCUSSION

In this experiment we aimed to investigate if the familiarity with the opponent and appraisal of challenge vs. threat moderated the T response to social competition in men. Participants assigned to the loser treatment significantly increased their T levels and no significant T change was observed in winners. This response to competition is specific to T, since for all other measured hormones no significant variation from pre- to post-competition levels was detected. These results for T cannot be fully explained by the biosocial model (Mazur, 1985) or the “challenge hypothesis” (Wingfield et al., 1990) since we have not found increased T in winners and decreased T in losers or a significant overall increase in T after the competition, respectively. The endocrine results for losers match previous findings with female samples using the same paradigm (Oliveira et al., 2013) and the T results in (Zilioli et al., 2014) with a female NTT competition that is only decided in the final trial (versus four trials in our experiment). Increases in T levels after losing a competition have been interpreted as an indicator of the individual’s motivation to keep engaged in competition in order to regain the status lost in the previous interaction (Mehta and Josephs, 2006; Oliveira et al., 2013; Zilioli et al., 2014). The hypothesis that the T changes occurring after the resolution of a competition are relevant for subsequent interactions, rather than for the current one, is supported by research in human and non-human animals showing that the social decision-making mechanisms in the brain are sensitive to changes in circulating levels of T (see Oliveira and Oliveira, 2014a). For example, the fear reducing properties of T (Hermans et al., 2006) may be of particular adaptive relevance to the individual in case of future agonistic interactions.

In our paradigm, the outcome of the competition was decided only in the last set of NTT trials and participants could monitor the score trial by trial. This may have influenced the losing participants’ engagement in the competition and evaluation of their capacity to compete against the winners and thus dispute their status in future interactions. However, the possible effect of these variables in the T response is undetermined and cannot be tested *post hoc* in the current experiment. Some support to this hypothesis can be found in a recent article by Zilioli et al. (2014). These authors argued that the uncertainty of the outcome generated by the alternation of wins and losses, ending with a close resolution of the contest, replicates an unstable status hierarchy and therefore the classical predictions of the biosocial model may not apply. In their experiments, the increase of T in losers and decrease in winners have been interpreted as indicators of competition seeking and competition avoidance, respectively (Zilioli et al., 2014). Together with the aforementioned research, our results suggest that men and women may exhibit the same endocrine response when losing a competition and provide preliminary evidence for a possible extension to males of the hypothesis proposed by Zilioli et al. (2014).

Since winners presented higher T than losers at the pre-competition measure, this experiment has limitations when it comes to findings related to the dynamics of T in male winners. For instance, we cannot exclude the possibility that the lack of a significant T increase in winners may be due to a ceiling effect. In fact before the competition subsequent winners had higher T levels than subsequent losers, but at the end of the competition T levels had increase in both groups and were not significantly different between them, yielding a significant increase from pre- to post-competition only in losers. Since winners and losers were experimentally assigned and randomized beforehand, the pre-competition difference between conditions cannot be attributed to *a priori* group differences or individual performance. Furthermore, experimenter bias was also controlled for, since the participants were free to choose their position in the competitive setting, thus self-selecting their experimental condition. Therefore, further research is required to clarify the inconclusive results for winners reported here.

Unlike a previous experiment with women in our lab (Oliveira et al., 2013), threat appraisal and opponent familiarity did not moderate the T increase found in men that lost the competition. Male losers also increased T and reported higher levels of threat than winners but neither variable was associated with opponent familiarity. Although previous research suggests a blunted or reduced T response in males when facing members of the ingroup (Wagner et al., 2002; Oxford et al., 2010; Trumble et al., 2012), this effect may reflect group processes that are not present in individual competition and therefore these previous findings may not be directly moderated or mediated by the effects of familiarity with the opponent as it was operationalized here.

Together, these results suggest that the psychological moderators of these T changes may differ between sexes or may have different weights in the interaction between cognitive processes and the T response. In the context of our experiment, the outcome elicited similar challenge and threat appraisals to those previously reported in females (Oliveira et al., 2013), however sex differences may exist concerning the importance of familiarity. This is congruent with previous research in which women were found to be more sensitive to familiarity than men, suggesting that this variable may have greater adaptive relevance for females (Deaner et al., 2007). Previous research with a male sample showed that the individual T levels were associated with the opponent’s self-efficacy, highlighting an evaluative process within the agonistic interaction in which the opponent’s characteristics relevant to the competition are assessed by the participants (van der Meij et al., 2010). Our results indicate that there may be sex differences in what is considered relevant in this evaluation. For instance the greater sensitivity to familiarity in women may explain the discrepancy of results using the same face to face competition. Furthermore, sex differences in psychological traits relevant to competition offer empirical support to this hypothesis. For example, a recent meta-analysis suggests that women are more sensitive to punishment and more averse to risk taking than men (Cross et al., 2011). Motivation toward competition is also different between the sexes. Men respond more strongly than women to intergroup conflict and therefore make more competitive choices in social dilemmas between groups than women (Wildschut et al., 2003; Van Vugt et al., 2007). Also, men are more motivated toward activities in which there are performance measures and opportunities to compete, when compared to women (Kilpatrick et al., 2006). These sex differences however may be strongly influenced by social hierarchy and context, since although men compete more than women in patriarchal societies, this pattern is reversed in matriarchal societies (Gneezy et al., 2009) and differences in motivation toward competition are attenuated or absent in same sex competitions (Niederle and Vesterlund, 2011). Although risk aversion does not directly explain differences in willingness to compete (Niederle and Vesterlund, 2011), it may still be an important factor influencing the appraisal process. Since most of the aforementioned findings result from competitions that used economic games as a competitive task, different paradigms are required to clarify the generalization of these attitudinal sex differences in competition and for the sex differences in relevant components for appraisal suggested in this article.

## Conflict of Interest Statement

The authors declare that the research was conducted in the absence of any commercial or financial relationships that could be construed as a potential conflict of interest.
